# LINE-1 cfDNA Methylation as an Emerging Biomarker in Solid Cancers

**DOI:** 10.3390/cancers16223725

**Published:** 2024-11-05

**Authors:** Ugur Gezer, Emre Özgür, Ebru E. Yörüker, Eleni Polatoglou, Stefan Holdenrieder, Abel Bronkhorst

**Affiliations:** 1 Department of Basic Oncology, Oncology Institute, Istanbul University, 34093 Istanbul, Türkiye; ugurd@istanbul.edu.tr (U.G.); emre.ozgur.86@istanbul.edu.tr (E.Ö.); akisik@istanbul.edu.tr (E.E.Y.); 2 Munich Biomarker Research Center, Institute of Laboratory Medicine, German Heart Center, Technical University Munich, 80636 Munich, Germanyholdenrieder@dhm.mhn.de (S.H.)

**Keywords:** cfDNA, ctDNA, liquid biopsy, clinical oncology, biomarkers

## Abstract

Epigenetic dysregulation is a hallmark of many human malignancies, with DNA methylation being a primary mechanism influencing gene expression and maintaining genomic stability. Genome-wide hypomethylation, characteristic of many cancers, is partly attributed to the demethylation of repetitive elements, including LINE-1, a prevalent non-LTR retrotransposon. The methylation status of LINE-1 is closely associated with overall genomic methylation levels in tumors. cfDNA comprises extracellular DNA fragments found in bodily fluids such as plasma, serum, and urine, offering a dynamic snapshot of the genetic and epigenetic landscape of tumors. This real-time sampling provides a minimally invasive avenue for cancer diagnostics, prognostics, and monitoring. The methylation status of LINE-1 in cfDNA has emerged as a promising biomarker, with several studies highlighting its potential in diagnosing and predicting outcomes in cancer patients. Recent research also suggests that cfDNA-based LINE-1 methylation analysis could serve as a valuable tool in evaluating the efficacy of cancer therapies, including immunotherapy. The growing clinical significance of cfDNA calls for a closer examination of its components, particularly repetitive elements like LINE-1. Despite their importance, the role of LINE-1 elements in cfDNA has not been thoroughly gauged. We aim to address this gap by reviewing the current literature on LINE-1 cfDNA assays, focusing on their potential applications in diagnostics and disease monitoring.

## Simple Summary

Cancer is often linked to changes in the regulation of genes, with DNA methylation playing a key role in controlling gene expression and maintaining stability. Many cancers show widespread loss of DNA methylation, particularly in repetitive elements like LINE-1, which can serve as an indicator of overall genomic health. Cell-free DNA (cfDNA), which is present in bodily fluids, offers a non-invasive way to track these changes and could be useful in diagnosing and monitoring cancer. Recent studies suggest that analyzing the methylation of LINE-1 in cfDNA could help predict disease outcomes and evaluate treatments like immunotherapy. This review aims to explore the current research on using LINE-1 methylation in cfDNA as a potential biomarker for cancer detection and therapy monitoring, addressing the need for further investigation into this promising approach.

## 1. Introduction

Cancer is a complex, multistep process that cannot be fully explained by genetic alterations alone. Growing evidence highlights the role of epigenetic modifications, which interact with genetic changes, environmental factors, and dietary habits to drive the development and progression of cancer [1,2]. Epigenetics refers to reversible chromatin modifications that occur independently of DNA sequence changes. Over the past 70 years, it has become a significant biological concept [3]. These modifications create an “epigenetic code” that regulates gene expression, dictating when and where genes are active, which plays a central role in development, health, and disease [4]. Key epigenetic mechanisms include DNA methylation, histone modifications, and non-coding RNAs, all of which are disrupted in cancer progression [1,2]. Understanding epigenetics and its interplay with genetic alterations is essential for developing more effective cancer prevention and treatment strategies.

DNA methylation, first identified in the 1940s, represents the earliest known epigenetic mark. Although its discovery dates back several decades, the role of DNA methylation in regulating gene expression was not recognized until the mid-1970s [5]. DNA methylation involves the transfer of a methyl group from S-adenosyl methionine to the fifth carbon of cytosine in the 5′-CG-3′ dinucleotide, forming 5-methylcytosine (5-meC). This process is catalyzed by DNA methyltransferases (DNMT), of which humans possess four types: DNMT1, DNMT2, DNMT3a, and DNMT3b. In eukaryotic genomes, the majority of 5′-CG-3 dinucleotides (60–90%) are methylated, with nonmethylated regions forming CpG islands, typically found in gene promotor regions and transcription start sites [6], which are critical for gene regulation. DNA methylation serves multiple purposes, including regulating gene expression, genomic imprinting, and maintaining genomic stability [7]. Unsurprisingly, aberrant DNA methylation is linked to various biological and physiological disruptions, contributing to aging and several diseases, most notably cancer. In cancer, DNA methylation is altered in two significant ways: global DNA hypomethylation and locus-specific hypermethylation. The latter results in increased methylation of CpG islands, leading to silencing of genes that control processes like cell cycle progression, DNA repair, and apoptosis, highlighting the importance of proper DNA methylation in maintaining cellular function and homeostasis [8].

Global DNA hypomethylation, characterized by a reduction in the 5-meC content across the genome, is a common epigenetic alteration in human tumors [9,10]. This hypomethylation is often associated with the demethylation of repetitive sequences in heterochromatic regions, including centromeric and pericentromeric repeats, LINE elements, and subtelomeric regions [11]. Such alterations in DNA methylation are closely linked to genomic instability in various cancer types [12,13,14,15]. Research using in vitro and animal models, along with primary tumor analysis, has consistently shown global DNA hypomethylation and genomic instability are associated with increased mutation rates and concomitant destabilization of heterochromatic regions leading to chromosomal rearrangements [16,17,18,19]. These findings suggest that the epigenetic deregulation observed in cancer cells undermines the protective role of DNA methylation in maintaining genomic integrity, thereby contributing to the progression and complexity of cancer.

LINE-1 methylation is an established, reliable indicator of global DNA hypomethylation in tumor tissues. Studies of cell-free DNA (cfDNA) have similarly confirmed that LINE-1 elements are hypomethylated in the plasma of cancer patients, indicating their potential as biomarkers. These findings also highlight the potential utility of LINE-1 methylation analysis in monitoring cancer therapy outcomes. This article reviews the clinical implications of LINE-1 methylation in cancer, with a special focus on the clinical utility of cfDNA in this context, summarizing the latest published data

## 2. LINE-1 Elements in the Human Genome

Repetitive DNA sequences represent patterns of DNA bases that occur in multiple copies throughout the genome [20], constituting approximately half of the human genome. These specialized sequences play critical roles that drive evolution, induce variation, and regulate gene expression. According to their distribution pattern in the human genome, repetitive DNA sequences are categorized into two groups: tandem repeats or satellites and interspersed elements [21]. Satellite sequences, also known as high-frequency repeats, are typically found in the pericentromeric and subtelomeric regions of human chromosomes and form constitutive blocks of heterochromatin, essential structural components of centromeres and telomeres [22]. The interspersed repeats represent transposable elements that, based on their mode of transposition (via DNA or RNA), are classified into two major types: DNA transposons and retrotransposons (RNA transposons). The retrotransposons are further composed of two subclasses: long terminal repeat (LTR) retrotransposons and non-LTR retrotransposons [23]. Among non-LTR retrotransposons that lack LTR, long interspersed elements (LINEs) and short interspersed nuclear elements (SINEs) are the two remaining active families with transpositional activity.

**Figure 1:**
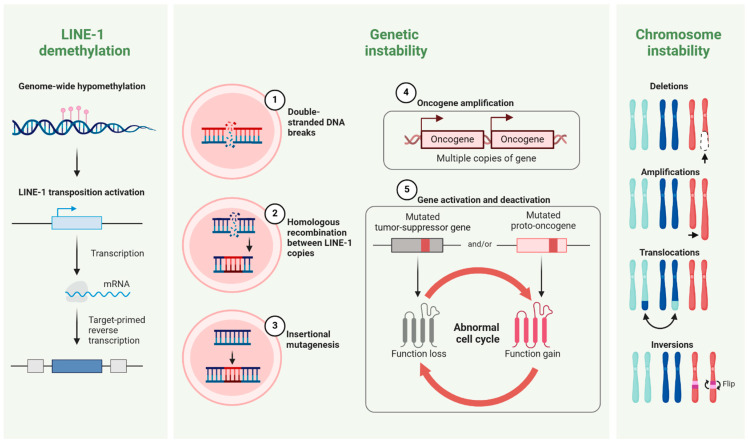
The role of LINE-1 demethylation in cancer. Genome-wide hypomethylation activates LINE-1 transposition (**left panel**), leading to double-stranded DNA breaks (1), homologous recombination between LINE-1 copies with similar base composition (2), and insertional mutagenesis (3). These events cause genetic instability, including oncogene amplification (4), gene activation or deactivation (5), and an abnormal cell cycle (**middle panel**). Chromosome instability (**right panel**) results in structural alterations such as deletions, amplifications, translocations, and inversions, further promoting tumorigenesis.

LINE-1, a member of the LINE family, is one of the most prevalent repeats, making up approximately 17% of the human genome [24]. LINE-1 elements encode RNA and proteins to make more copies of themselves, which can then integrate into new genomic loci. This process is repressed in most normal cells via methylation, whereas LINE-1 derepression is a hallmark of many human tumors [25]. As LINE-1 derepression could cause cellular damage, many studies have provided evidence that the transpositional activity of LINE-1 may contribute to the development of human cancers (oncogenic pathways summarized in Figure 1) [26]. It has been shown that homologous recombination between two LINE-1 copies with similar base compositions could activate chromosomal rearrangements such as large inversions and duplications [14,27]. In cancer cells, LINE-1 retrotransposons could also induce insertional mutagenesis, leading to the loss of gene function [28,29]. LINE-1-induced deletions can occasionally lead to removing tumor-suppressor genes or amplifying oncogenes, rendering an oncogenic function to the LINE-1 element.

## 3. LINE-1 Methylation Status as a Marker of Global DNA Hypomethylation in Cancer

The LINE-1 activity in most somatic cells is generally suppressed by CpG methylation within its internal promoter [30,31]. However, in tumor cells, this methylation is often lost, leading to increased retrotransposition [32]. The methylation status of CpG sites within the LINE-1 element is strongly correlated with global genomic DNA methylation [33], making LINE-1 hypomethylation a reliable surrogate marker for assessing overall DNA hypomethylation, a phenomenon commonly observed in aging and cancer [34,35,36]. Distinct differences in LINE-1 methylation between normal and tumor tissues are well documented, with consistent hypomethylation identified across multiple cancer types, including breast, lung, colon, gastric, ovarian, and bladder cancers, hepatocellular carcinoma, gastrointestinal stromal tumor, extra-hepatic cholangiocarcinoma, and chronic lymphocytic leukemia [37,38,39,40,41,42,43,44,45]. In breast cancer, LINE-1 hypomethylation is notably associated with the HER-2-enriched subtype [46] and correlates with tumor histological grade, indicating a potential role in tumor progression and prognosis [38,40,43,47,48]. The broader implications of LINE-1 methylation as a diagnostic and prognostic biomarker in cancer have been comprehensively reviewed in recent literature [49].

Patterns of LINE-1 methylation in peripheral blood cells have also been explored as a potential biomarker in cancer diagnostics. A study on breast cancer provided a comparative analysis of LINE-1 methylation in DNA extracted from both tumor tissue and peripheral blood. The results demonstrated significant hypomethylation in tumor tissue, while no comparable changes were observed in blood cells. These findings suggest that LINE-1 hypomethylation in peripheral blood is not a reliable marker for early breast cancer detection, underscoring this epigenetic modification’s tissue-specific nature [50]. Conversely, another study reported that LINE-1 hypomethylation, along with the methylation of satellite 2 repeat in blood cells, correlated with an increased risk of advanced gastric lesions and subsequent development of gastric cancer [51]. This suggests that LINE-1 methylation patterns in peripheral blood may have the potential to be biomarkers in gastric cancer progression. In prostate cancer, findings have been inconsistent. Han et al. found no association between LINE-1 methylation in blood cells and the risk of aggressive prostate cancer or biochemical recurrence following treatment in a cohort of 795 patients [52]. However, Xu et al. reported that lower LINE-1 methylation levels were linked to an increased risk of biochemical recurrence (BCR) and shorter BCR-free survival times [53], highlighting the complexity and potential context-dependent nature of LINE-1 methylation as a prognostic marker in prostate cancer. In head and neck cancer, one study demonstrated that reduced LINE-1 methylation levels in blood cells could differentiate patients from controls with 100% sensitivity and specificity [54], suggesting significant diagnostic potential of LINE-1 methylation in blood cells in head and neck cancer.

## 4. Clinical Significance of Cell-Free DNA in Oncology

Molecular profiling of tumor specimens currently represents the standard in cancer diagnosis, aiding in the detection of cancer-specific mutations, tumor classification, and guiding treatment decisions [55]. However, despite its widespread use, this approach is limited by inherent biological variability and technical challenges associated with tumor tissue analysis. The invasive nature precludes serial analyses, making real-time monitoring of cancer progression and therapy response unfeasible. Additionally, limited tissue availability often restricts the scope of molecular testing, compromising diagnostic accuracy. Furthermore, the inherent inter- and intratumor heterogeneity introduces substantial genomic variability, leading to the presence of distinct subclones with varying drug sensitivities. As a result, a single tumor tissue analysis may fail to capture the full spectrum of tumor biology, potentially overlooking critical insights into disease progression and treatment resistance [56]. These challenges underscore the need for more advanced and complementary approaches to provide a more comprehensive and dynamic understanding of tumor biology, thereby improving the accuracy and reliability of cancer diagnostics and therapeutics. Here, liquid biopsy is a real alternative to tissue biopsy with many advantages. As liquid biopsy samples are obtained through minimally invasive procedures, e.g., through a blood draw, longitudinal follow-up of genetic mutations in cancer therapy could facilitate real-time monitoring of treatment outcomes and eventually monitoring the emergence of mutations underlying the development of drug resistance [57,58].

cfDNA comprises extracellular DNA fragments found in bodily fluids such as plasma, serum, urine, saliva, and bronchial lavage. Since the first detection of cfDNA in serum from cancer patients [59], scientific interest in this field has been growing. It is now evident that most of the cfDNA fragments released into serum/plasma in healthy individuals stem from cells of the hematopoietic system [60]. In cancer patients, however, cfDNA contains both tumoral and non-tumoral DNA wherein the tumor-derived fraction represents a small portion and may bear tumor-specific mutations, known as circulating tumor DNA (ctDNA). The ctDNA fraction of plasma DNA may vary depending on the cancer type and tumor load, as ctDNA levels are generally higher in patients with advanced disease than in early-stage cancers [61,62].

cfDNA exhibits a size distribution predominantly centered around 167 bp, corresponding to the length of DNA wrapped around a nucleosome. This characteristic non-random fragmentation pattern arises from endonucleases’ selective cleavage of the internucleosomal regions [63]. In contrast, longer DNA fragments, often originating from necrotic cell death, are also co-present in circulation [64,65,66,67,68,69]. Notably, tumor-derived cfDNA exhibits a higher degree of fragmentation compared to non-tumor cfDNA, with significant enrichment of short fragments (<145 bp) in plasma, as demonstrated by several studies [70,71,72,73]. This fragmentation profile provides a potential biomarker for distinguishing tumor-derived cfDNA from non-tumor DNA in liquid biopsies.

cfDNA analysis is increasingly seen as a key tool in cancer diagnosis, treatment, and clinical follow-up. The FDA’s 2016 approval of a liquid biopsy for *EGFR* mutations in non-small cell lung cancer accelerated its use in clinical practice [74]. cfDNA profiling is now integral for detecting tumor mutations, guiding therapy, and monitoring treatment response [75]. While ctDNA-based liquid biopsies have advanced the personalization of cancer therapy, the clinical application of liquid biopsy approaches that include DNA methylation is still in its early stages [76]. Although DNA methylation holds significant potential as a biomarker for various cancer indications [77,78,79], its clinical application requires further validation. Recent developments in cfDNA-based methylation analysis have created several commercial platforms designed for cancer detection with varying sensitivity and specificity. These platforms target a range of cancers, including lung, colon, liver, and bladder, as well as multi-cancer screening. The opportunities and limitations of methylation-based liquid biopsies in companion diagnostics were highlighted in a recent review [80]. Here, we focus on the clinical relevance of LINE-1 methylation in cfDNA and the methodologies employed for its detection.

## 5. Methods Used for LINE-1 Methylation Analysis in Cell-Free DNA

**Figure 2:**
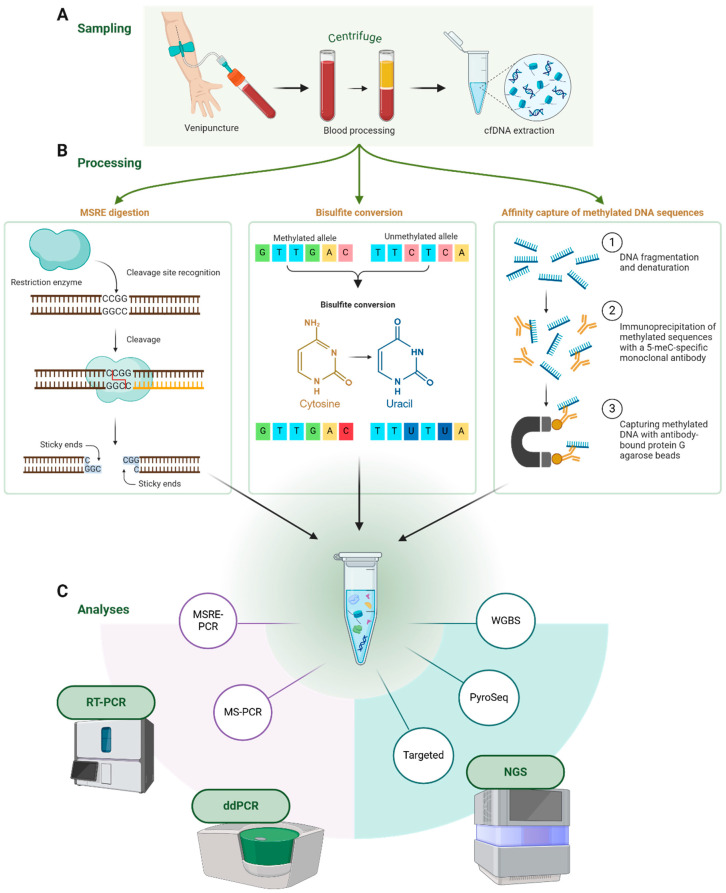
Methods for characterizing LINE-1 methylation in cell-free DNA (cfDNA). (**A**) Blood is collected and processed, and cfDNA is extracted. (**B**) The cfDNA is then processed using one of three methods: (i) methylation-sensitive restriction enzyme (MSRE) digestion, which cleaves unmethylated DNA to detect methylation at specific CpG sites; (ii) bisulfite conversion, converting unmethylated cytosine into uracil, distinguishing methylated from unmethylated alleles for precise analysis; and (iii) affinity capture of methylated DNA, isolating methylated sequences for genome-wide analysis. (**C**) Processed DNA is analyzed using techniques like PCR, pyrosequencing, or next-generation sequencing for targeted or genome-wide methylation profiling.

cfDNA methylation profiling has rapidly evolved, transitioning from early studies focused on identifying and quantifying methylated CpG sites within specific genes to broader applications facilitated by technological advancements [81]. The introduction of microarray hybridization and high-throughput sequencing has enabled genome-wide mapping of DNA methylation patterns, significantly expanding the scope of research. Various methods are now available for analyzing DNA methylation, each with distinct advantages depending on the biological questions being addressed. Key techniques include (i) a methylation-sensitive restriction enzyme (MSRE) digestion-based analytical technique followed by PCR (MSRE-PCR) or sequencing, (ii) techniques based on bisulfite conversion of DNA followed by PCR (MS-PCR) or sequencing, (iii) affinity capture of methylated DNA followed by PCR or sequencing, and (iv) liquid chromatography linked with tandem mass spectrometry (LC-MS/MS) [82]. These methodologies (summarized in Figure 2), particularly the first three or their modified versions, have been integral in analyzing LINE-1 methylation in cfDNA, highlighting their utility in targeted and global methylation studies. The choice of technique should be guided by the specific research goals, balancing sensitivity, specificity, and the scale of analysis.

### 5.1. MSRE Digestion-Based Analysis of DNA Methylation

The discovery of methylation-sensitive restriction enzymes (MSREs), a class of restriction enzymes that specifically cleave DNA at unmethylated recognition sites, has advanced the study of DNA methylation. MSREs such as MspI and HpaII cleave DNA strands at unmethylated CpG dinucleotides, allowing researchers to assess DNA methylation status based on the presence or absence of amplification in subsequent PCR. This method provides a straightforward approach to detect site-specific DNA methylation [83]. Recent advancements include the integration of digital droplet PCR (ddPCR) [84] and computational tools that reconstruct CpG site information from next-generation sequencing (NGS) data post-MSRE digestion, thereby expanding the utility of MSRE-based techniques [85]. While MSRE-based methods do not quantify methylation levels, they remain valuable for identifying methylation patterns across different genes and genomic regions [86]. Over the past two decades, MSRE-PCR has been extensively applied to assess gene-specific methylation, particularly in cancer research, where it has been used to evaluate hypermethylation of target genes in cfDNA [87,88,89]. Additionally, MSRE-PCR has been applied to assess the methylation status of repetitive elements, such as LINE-1 sequences, in cfDNA from cancer patients [90,91,92].

### 5.2. Bisulfite Conversion-Based Methods

Bisulfite conversion-based analysis of DNA methylation is considered the gold standard of this field [93]. In this technique, the first step is the treatment of input DNA with sodium bisulfite, which causes the deamination of unmethylated cytosine residues in uracil and, finally, in thymine through subsequent PCR. Methylated cytosine residues remain unaffected through bisulfite treatment [94], requiring specifically designed primers for converted and unaffected sequences in the subsequent amplification step, called methylation-specific PCR (MS-PCR). Subsequent MS-PCR or targeted sequencing will facilitate gene-specific methylation analysis, while whole genome bisulfite sequencing will be more informative in establishing genome-wide mapping of methylated sites. Even if widely used in epigenetic research, the degradation of significant levels of input DNA represents the main shortcoming of the bisulfite conversion-based technique [95]. MSRE bisulfite sequencing represents a variant of bisulfite sequencing [96]. Most studies evaluating LINE-1 methylation status in cfDNA of cancer patients employed MS-PCR [97,98,99,100,101,102,103,104,105]. A technique based on combining bisulfite conversion with restriction enzyme digestion (COBRA) followed by PCR was also used in the methylation analysis of LINE-1 in cfDNA in cancer patients [106].

Pyrosequencing is a sequencing-by-synthesis technique that enables rapid and accurate quantification of sequence variation. LINE-1 pyrosequencing after bisulfite treatment is a widely used method for the estimation of global DNA methylation [107,108,109]. Many studies also use it to assess the LINE-1 methylation status in cfDNA in cancer patients [110,111,112,113,114,115]. Aparicio et al. demonstrated that LINE-1 bisulfite pyrosequencing was superior to other DNA methylation analyses, such as methylation-sensitive single-nucleotide primer extension, COBRA, and MethyLight regarding the assay variability and the signal-to-noise ratio and proved to be a feasible assay to track DNA methylation changes in plasma of cancer patients treated with DNA methylation inhibitors [110]. In a study with bladder cancer patients, DNA methylation was analyzed in formalin-fixed, paraffin-embedded tissue, serum, buffy coat, and buccal cells by bisulfite pyrosequencing, and serum samples had the highest average levels of LINE-1 methylation [115].

Szigeti et al. conducted a comprehensive analysis of how various biological and experimental variables influence LINE-1 bisulfite pyrosequencing in colorectal tissues (n = 222), buffy coat (n = 39), and plasma samples (n = 9) from both healthy individuals and patients with colorectal tumors. Their findings indicate that formalin-fixed, paraffin-embedded biopsies consistently exhibit lower LINE-1 methylation levels than freshly frozen tissues, with long-term DNA storage resulting in higher methylation levels. In blood collection tubes with preservatives, methylation levels in cfDNA and buffy coat were significantly higher compared to K3EDTA tubes. Interestingly, while storage conditions, such as temperature (RT and 4 °C) and duration (0, 3, and 6 h) had no measurable impact on LINE-1 methylation level in both buffy coat and cfDNA in conventional K3EDTA and cfDNA collection tubes [111], contrasting evidence from Van Paemel et al. suggest that longer storage times (72 h) in EDTA tubes can increase methylation in cfDNA [116]. This difference may be the result of only a small number of samples analyzed. Nevertheless, this discrepancy emphasizes the importance of standardizing preanalytical conditions to ensure consistent and reliable LINE-1 methylation analysis across studies.

### 5.3. Affinity Capture of Methylated DNA Sequences 

Affinity-based enrichment of methylated DNA is a relatively widely used approach to enrich the methylated regions of genomic DNA. It is based on the immunoprecipitation of methylated DNA using specific antibodies for 5-meC [86]. The main steps of this approach include (i) fragmentation of denatured DNA, either mechanically (e.g., sonication) or enzymatically; (ii) immunoprecipitation of methylated sequences with a monoclonal antibody specific for 5-meC, (iii) capturing antibody-bound methylated DNA fragments by protein G agarose beads, and (iv) elution of methylated fragments from beads. In the case of cfDNA, fragmentation of DNA is not necessary in the first step. Enriched fragments can undergo a methylation analysis of individual CpG sites by PCR or be subjected to next-generation sequencing to outline the distribution of methylated CpG sites across the whole genome. Methyl capture assay coupled with quantitative PCR and targeted sequencing was employed in one study to analyze cfDNA-based LINE-1 methylation status in lung cancer patients [117].

## 6. Clinical Significance of LINE-1 Methylation Detection in cfDNA in Solid Cancers

Of the 19 studies that investigated LINE-1 methylation in cfDNA, nearly half (9) focused on its potential as a diagnostic biomarker, while six examined its prognostic implications. The remaining four studies explored its utility in monitoring therapeutic response. The cancers evaluated in these studies spanned a range of types, including breast, lung, prostate, colorectal, and gastric cancers, hepatocellular and esophageal carcinomas, and malignant melanoma (Table 1).

### 6.1. Diagnostic Potential of LINE-1 Methylation in cfDNA in Solid Cancers

Lee et al. investigated the diagnostic performance of LINE-1 hypomethylation in plasma cfDNA for breast cancer, a leading cause of cancer-related mortality in women worldwide despite advancements in early diagnosis and treatment [90]. Using MSRE-PCR, they demonstrated that two human breast cancer cell lines (e.g., MCF-7 and MDA-MB-231) exhibit significant hypomethylation at CpG islands within the 5′ UTR of LINE-1 compared to the non-cancerous cell line MCF 10a. Furthermore, LINE-1 methylation was found to be decreased in both canine and human breast tumors. The study extended these findings to clinical samples, revealing significantly lower LINE-1 methylation levels in cfDNA from breast cancer patients (n = 26) compared to healthy controls (N = 36). The diagnostic performance, as reflected by an area under the receiver operating curve (AUC) of 0.7808 (*p* = 0.01), suggests that LINE-1 hypomethylation in cfDNA possesses moderate discriminatory power for detecting breast cancer [90].

A subsequent study involving 64 breast cancer patients and 64 healthy controls confirmed these results, showing significantly lower LINE-1 methylation in breast cancer patients (*p* < 0.01), with an AUC of 0.890, sensitivity of 78%, and specificity of 83% at a cut-off value ≤ 90 [97]. Additionally, similar patterns of decreased LINE-1 methylation in cfDNA were observed in lung cancer patients (n = 64) with an AUC of 0.848, sensitivity of 75%, and specificity of 87.50% at the cut-off point of ≤90. 

These findings are extended through previous investigations by Gainetdinov and colleagues, who explored LINE-1 methylation levels in the cell-surface-bound (csb) fraction of cfDNA. Their cohort included 59 patients with non-small lung cancer and 47 healthy donors of comparable age. Using an affinity capture assay coupled with qPCR, they showed that a substantial fraction of cfDNA and RNA is bound to the surface of blood cells and further demonstrated a notable reduction in LINE-1 promoter methylation in lung cancer patients, with an AUC of 0.69 (*p* = 0.0012). Additionally, targeted sequencing revealed a higher frequency of hypomethylation in the human-specific L1Hs subfamily among cancer patients [117]. In a subsequent study, this group examined LINE-1 methylation in a more diverse cohort, including controls with bronchitis or chronic obstructive pulmonary disease (COPD). LINE-1 methylation was quantified using MS-PCR, with results expressed as genome equivalents per milliliter (ge/mL). The findings revealed a significantly lower mean concentration of LINE-1 in lung cancer patients (97 ge/mL) compared to healthy individuals (344 ge/mL), patients with bronchitis (310 ge/mL), and those with COPD (236 ge/mL). These results suggest that cfDNA-based LINE-1 methylation analysis holds promise as a differential diagnostic tool, particularly in distinguishing lung cancer from inflammatory lung diseases [98].

Nagai et al. evaluated LINE-1 hypomethylation levels in plasma cfDNA of patients with colorectal cancer (CRC). In a cohort including 114 patients and 53 healthy donors, they quantified LINE-1 methylation using absolute quantitative MS-PCR, and a LINE-1 hypomethylation index (LHI) was derived by dividing the sum of unmethylated copies by methylated and unmethylated copies. Elevated LHI values, indicating increased hypomethylation, were associated with larger tumors (≥6.0 cm), a higher number of positive lymph nodes (≥2), and distant metastasis. This suggests that cfDNA LHI could be a marker of disease progression. Moreover, early-stage and advanced-stage CRC patients exhibited significantly higher cfDNA LHI than healthy controls. The discriminatory power of cfDNA LHI was notable, with AUC values of 0.79 and 0.83 for stage I/II and stage III/IV patients, respectively [99], indicating its potential utility in CRC diagnosis. Further supporting these findings, a recent study demonstrated reduced LINE-1 methylation in both plasma and tissue samples from patients with colon adenomas and CRC, compared to normal tissues and healthy controls. At a cut-off value of 80.0%, plasma LINE-1 hypomethylation differentiated adenomas from controls with a sensitivity of 67% and a specificity of 90% (AUC = 0.8) [112].

Delgado-Cruzata et al. conducted a case-control study investigating the methylation status of LINE-1, *p16*, *GSTP1*, and *APC* genes in patients with stage II/III prostate cancer, the most common malignancy in men. The study measured methylation levels in paired tissue samples (24 pairs) and plasma samples (27 patients, 24 controls) using quantitative pyrosequencing. The study showed concordance between tissue and plasma methylation levels for LINE-1 and *GSTP1*. Notably, LINE-1 methylation was significantly lower in tumor tissues compared to adjacent normal tissues and in the cfDNA of patients relative to healthy controls. Moreover, plasma LINE-1 methylation correlated with higher Gleason scores (≥7), indicating its potential as a marker for aggressive prostate cancer [113].

In a separate study by Boldrin and colleagues [91], the methylation status of LINE-1 in cfDNA was assessed in patients with esophageal adenocarcinoma (n = 19) and Barrett’s esophagus (n = 2) using MSRE-PCR. The analysis focused on two methylation sites (CCGG) placed in the LINE-1 promoter, nucleotides 36–39 (site A) and nucleotides 305–308 (site B). Hypomethylation at site A was observed in over 60% of patients, with a median methylation level of 67% compared to 100% in fully methylated constitutive DNA (*p* = 0.0001). Site B exhibited less pronounced hypomethylation. Longitudinal analysis in patients with Barrett’s esophagus suggested a possible link between LINE-1 methylation status in cfDNA and the progression to esophageal adenocarcinoma, highlighting the potential utility of LINE-1 methylation analysis in patient monitoring.

The collective findings from these studies underscore the significant potential of LINE-1 hypomethylation in cfDNA as a minimally-invasive biomarker for the early detection, diagnosis, and monitoring of various cancers, including breast, lung, colorectal, prostate, and esophageal cancers. The consistent evidence across multiple cancer types highlights the discriminatory power of LINE-1 hypomethylation in differentiating cancer patients from healthy individuals and those with benign conditions. However, while the diagnostic performance metrics, such as AUC values, sensitivity, and specificity, are promising, further research involving larger and more diverse cohorts is essential to validate these findings and establish the clinical utility of LINE-1 hypomethylation in routine cancer screening and monitoring. Future studies should also explore the integration of LINE-1 methylation analysis with other biomarkers and diagnostic tools to enhance the accuracy and reliability of cancer detection and prognosis.

### 6.2. Prognostic Value of LINE-1 Methylation in cfDNA

The prognostic implications of LINE-1 methylation in cfDNA and its associated long fragment concentration were assessed in gastric cancer patients undergoing curative surgery [92]. The study involved 99 patients, with blood samples collected at baseline and one-month post-surgery or before chemotherapy in those receiving adjuvant treatment. LINE-1 methylation was quantified using a modified MSRE-PCR technique, termed HELP (HpaII tiny fragment Enrichment by Ligation-mediated PCR). Patients presenting low baseline LINE-1 methylation levels exhibited reduced overall survival (OS) compared to those with higher methylation levels. Among the surgical cohort (n = 90), both recurrence-free survival and OS were notably poorer in patients with lower LINE-1 methylation prior to surgery (*p* = 0.08 and *p* = 0.11, respectively), suggesting that pre-surgical LINE-1 hypomethylation serves as a negative prognostic factor for gastric cancer.

In a 2007 study, Tangkijvanich et al. investigated the prognostic significance of LINE-1 methylation in patients with hepatocellular carcinoma (HCC, n = 85), in comparison with patients with cirrhosis (n = 73), individuals carrying hepatitis B virus (n = 20) and healthy subjects (n = 30). LINE-1 methylation status was evaluated by COBRA followed by PCR in serum samples. The results demonstrated a significantly higher percentage of hypomethylated LINE-1 HCC patients compared to controls (*p* < 0.001), with this hypomethylation correlating with larger tumor size and more advanced disease stages. Notably, LINE-1 hypomethylation emerged as an independent prognostic marker for overall survival in HCC patients [106]. Building on these findings, a subsequent study in 2011 analyzed LINE-1 methylation in sera of patients of 50 HCC patients, 20 cirrhosis patients, 20 individuals with chronic hepatitis C, and 10 healthy subjects, utilizing MS-PCR. Consistent with the earlier study, LINE-1 hypomethylation in serum was significantly associated with poor survival in HCC patients [102].

In a prospective study involving 172 HCC patients, the researchers investigated the prognostic significance of LINE-1 methylation levels in plasma and blood leukocytes. Methylation at three CpG sites was quantified using bisulfite pyrosequencing, and the average methylation across these sites was calculated. Patients with LINE-1 hypomethylation (<70%) exhibited significantly reduced OS compared to those with higher methylation levels (≥70%), with a hazard ratio of 1.77. Notably, the combination of low LINE-1 methylation and reduced plasma folate levels was associated with an even greater risk of poor survival (HR = 3.36). In contrast, LINE-1 methylation in leukocytes showed no correlation with patient prognosis. This data suggests that plasma LINE-1 hypomethylation, particularly when coupled with lower folate levels, serves as an unfavorable prognostic indicator [114].

In a separate study by Liu et al., the prognostic utility of LINE-1 hypomethylation combined with *RASSF1A* gene hypermethylation in sera was evaluated for predicting early recurrence post-curative surgery in HCC patients. LINE-1 hypomethylation was detected in 67% of patients (70/105), while *RASSF1A* promoter hypermethylation was present in 73% of HCC samples. These methylation changes were absent in control samples. LINE-1 hypomethylation was significantly associated with Hepatitis B virus surface antigen (HBsAg) positivity (*p* = 0.009), larger tumor size (*p* = 0.001), and elevated alpha-fetoprotein levels (*p* < 0.001). Survival analysis revealed that LINE-1 hypomethylation strongly correlated with poorer disease-free survival (*p* = 0.002) and OS (*p* = 0.0123). Moreover, the combination of LINE-1 hypomethylation and *RASSF1A* promoter hypermethylation effectively predicted early recurrence and was linked to poor prognosis in HCC patients post-resection [103].

Based on previous findings that tumoral methylation of LINE-1 and the absent in melanoma-1 (*AIM1*) gene was associated with progression and disease outcome in melanoma, Hoshimoto and colleagues evaluated the prognostic significance of both markers in paraffin-embedded archival tissues (n = 133) and sera (n = 56) of melanoma patients. Serum levels of unmethylated LINE-1 were found to be significantly higher in patients with advanced (stage III and IV) compared with healthy controls (*p* = 0.022). Furthermore, the presence of either serum unmethylated LINE-1 or methylated *AIM1* correlated with reduced survival (*p* = 0.0009), indicating the prognostic utility of these markers for predicting disease progression and patient outcomes in melanoma [105].

The studies demonstrate that LINE-1 hypomethylation in cfDNA is a significant prognostic marker across various cancers, including gastric, HCC, and melanoma. In gastric cancer, lower LINE-1 methylation levels correlate with poorer overall survival, while in HCC, LINE-1 hypomethylation is consistently associated with worse survival outcomes, larger tumor size, and advanced disease stages. Additionally, combining LINE-1 hypomethylation with other markers, such as *RASSF1A* hypermethylation in HCC or *AIM1* methylation in melanoma, enhances prognostic accuracy and predicts early recurrence post-surgery. These findings highlight the clinical relevance of LINE-1 hypomethylation as an independent prognostic marker. Future research should focus on standardizing detection methods and validating these results in larger cohorts to facilitate the integration of LINE-1 methylation analysis into clinical practice.

### 6.3. Significance of LINE-1 Methylation in cfDNA in the Assessment of Cancer Therapy

Ponomaryova et al. (2017) investigated the role of LINE-1 methylation in predicting treatment response in lung cancer patients undergoing neoadjuvant chemotherapy followed by surgical resection. The study involved a cohort of 16 patients, with LINE-1 methylation levels assessed in plasma cfDNA and csb-cfDNA using MS-PCR. A normalization strategy involving a methylation-independent LINE-1 region was employed to quantify methylation changes. Notably, LINE-1 methylation in the csb-cfDNA fraction increased two-fold post-chemotherapy and three-fold post-surgery, suggesting a dynamic response to treatment. This methylation change was more pronounced in patients with squamous cell carcinoma compared to those with adenocarcinoma. These findings highlight the potential of csb-cfDNA LINE-1 methylation as a biomarker for monitoring therapeutic efficacy in lung cancer [104].

In a recent study by the same group, the methylation status of LINE-1, *SEPTIN9*, and *IKZF1* genes in cfDNA and csb-cfDNA was determined in rectal cancer, focusing on their potential as biomarkers for therapy response and relapse prediction. Blood samples were collected at multiple time points: before treatment, post-preoperative chemotherapy, 10–15 days after surgery, and quarterly during a 12-month follow-up. Methylation levels were quantified using MS-PCR. This revealed a significant increase in LINE-1 methylation in csb-cfDNA following chemotherapy (1.6-fold) and tumor resection (3-fold) compared to baseline. Among patients who experienced relapse (n = 5), there was a marked increase in *SEPTIN9* and *IKZF1* methylation, coupled with a decrease in LINE-1 methylation, within two weeks post-surgery. In contrast, patients without relapse (n = 20) exhibited no significant changes in methylation levels throughout the follow-up period. These findings suggest that LINE-1 and other gene methylations in cfDNA may serve as valuable biomarkers for monitoring therapeutic efficacy in early relapse detection rectal cancer patients [100]. Complementarily, Barták et al. employed a liquid biopsy approach to monitoring the therapy response in CRC, assessing LINE-1 methylation, *SFRP2* and promotor methylation, the plasma homocysteine levels in patients with non-metastatic and metastatic CRC receiving chemotherapy. They found that cfDNA concentrations were higher in patients with progressive and recurrent disease compared to those in remission or with stable disease (*p* < 0.05). Interestingly, global methylation via LINE-1 declined (mean 75.5% vs. 68.2%), while gene promoter methylation and homocysteine levels increased in patients with progressive disease. In cases with remission, opposite changes were observed [101]. Both studies contribute to the growing evidence that cfDNA methylation profiles can serve as effective biomarkers for monitoring treatment response and detecting early signs of relapse in colorectal cancer, offering a minimally invasive tool for personalized patient management.

In another study, the researchers focused on the potential role of LINE-1 hypomethylation as a predictive marker for the efficacy of immune checkpoint blockade (ICB) therapy in cancer treatment. Although ICB therapy has shown promise across various cancers, its long-term effectiveness is observed in only a minority of patients. Conventional markers—tumor mutational burden (TMB), PD-L1 expression, and defects in the mismatch repair deficiencies have demonstrated limited predictive accuracy and clinical utility [119]. Building on prior findings that the hypomethylation of evolutionarily young LINE-1 elements in tumor tissues can outperform TMB in predicting ICB therapy benefits in lung cancer and melanoma [120], Kim et al. explored the utility of LINE-1 hypomethylation in cfDNA for predicting ICB responses. Their study employed a deep targeted sequencing method, termed iMethyl, to analyze methylation patterns of young LINE-1 elements in cfDNA and tissue samples from lung (cfDNA n = 167; tissue n = 137) and breast cancer (cfDNA n = 91; tissue n = 50) patients. Tissue-based methylation analysis provided superior predictive power for ICB response compared to PD-L1 expression and TMB and, importantly, was independent of tumor purity. Using matched samples of breast cancer patients with progression (n = 44), the authors showed a significant methylation loss at most LINE-1 target sites in plasma during tumor progression, and the iMethyl-liquid assay was capable of predicting disease progression under ICB therapy. Further investigation in a subset of patients revealed that early changes in cfDNA LINE-1 methylation, specifically progressive hypomethylation in non-responders and hypermethylation in responders, could be detected within the first weeks of treatment. This finding underscores the potential of cfDNA LINE-1 methylation as a minimally-invasive marker for monitoring early therapeutic responses to ICB [118].

These studies highlight the potential of LINE-1 methylation in cfDNA as a valuable biomarker for monitoring cancer therapy and predicting disease progression. Dynamic changes in LINE-1 methylation, observed in response to treatments like chemotherapy and surgery, suggest its utility in real-time assessment of therapeutic efficacy. Moreover, differential methylation patterns in patients who relapse versus those in remission underscore its potential for early detection of recurrence. The use of LINE-1 hypomethylation as a predictive marker for ICB therapy also shows promise, offering superior predictive power compared to traditional markers. Early detection of changes in cfDNA LINE-1 methylation during ICB therapy could enable more personalized treatment approaches.

The analysis of LINE-1 methylation in cfDNA holds promising potential for cancer management across different disease indications and many types of cancer. However, like most cfDNA-based assays, LINE-1 methylation analysis has not yet reached the high sensitivity and specificity needed for regular clinical use, mainly because of biological and technical complexities that make accurate detection challenging. Ideally, a biomarker should demonstrate both high sensitivity and specificity, but few cancer biomarkers meet these standards. This limitation is largely due to genetic and epigenetic differences within and between tumors, which lead to varying concentrations of biomarkers in cfDNA. LINE-1 methylation faces similar issues, along with other biological factors such as the extent of cell death, levels of cfDNA fragmentation, clearance rates from the bloodstream, and differences among individual patients that further impact the sensitivity and specificity of cfDNA markers (reviewed in [57]). In addition to these biological complexities, cfDNA-based epigenetic profiling also faces significant preanalytical, technical, and analytical challenges, with notable issues like similar or identical epigenetic modifications that occur in both normal biological processes and disease states, making it difficult to distinguish disease-specific markers. Additionally, random changes in specific epigenetic biomarkers can introduce noise, while biases in various detection methods can alter the observed epigenetic profile and complicate accurate analysis. Current detection technologies may also lack the ability to identify small methylation changes in cfDNA, which limits the diagnostic power of these tests. Some NGS diagnostic providers use various epigenetic markers to report tumor fraction percentages based on DNA methylation in their liquid biopsy results. Direct comparison studies are needed to determine if LINE-1 methylation performs as well as or better than these other markers. Research suggests that using multiple markers together, rather than relying on a single one, generally improves diagnostic accuracy for cancer detection. Therefore, combining LINE-1 methylation with other epigenetic biomarkers could enhance the sensitivity and specificity of cfDNA-based tests. While the clinical value of these cfDNA features still needs further validation, existing evidence suggests that examining these epigenetic characteristics could help drive the development of more effective cfDNA-based assays. It will be essential to address the technical and biological challenges involved to achieve this.

## 7. Conclusions

Liquid biopsies represent a significant advancement in biomarker research, providing a minimally invasive method to analyze circulating tumor cells, cfDNA, and exosomes. This approach has paved the way for precision oncology, particularly through the detection of tumor-specific mutations in cfDNA, which has facilitated personalized cancer treatments. In addition to DNA mutations, epigenetic alterations, such as DNA methylation, offer promising biomarker potential. LINE-1 hypomethylation, a marker of genomic instability, has emerged as a key player in cancer diagnosis and prognosis. Numerous studies have demonstrated decreased LINE-1 methylation in cfDNA across various cancers, including breast, lung, colorectal, prostate, and esophageal cancers, with strong diagnostic accuracy. Moreover, LINE-1 hypomethylation has been consistently associated with poor survival outcomes, advanced disease stages, and larger tumors in cancers like HCC and gastric cancer. Combining LINE-1 hypomethylation with other epigenetic markers, such as *RASSF1A* hypermethylation in HCC or *AIM1* methylation in melanoma, has further enhanced prognostic accuracy and predicted early recurrence after surgery. Beyond diagnostics, LINE-1 methylation is showing potential for monitoring cancer therapies. Dynamic changes in LINE-1 methylation have been observed during treatments such as chemotherapy, surgery, and immunotherapy, indicating its value in assessing treatment efficacy in real-time. Its ability to predict relapse and disease progression is particularly promising, and in ICB therapy, LINE-1 hypomethylation has demonstrated superior prognostic power compared to traditional markers. Early detection of changes in LINE-1 methylation during ICB therapy could support more personalized treatment approaches. However, while the potential of LINE-1 hypomethylation as a biomarker is clear, larger and more diverse studies are needed to validate these findings and establish its clinical utility in routine cancer screening, diagnosis, and treatment monitoring. Preanalytical optimization, standardization, and the development of robust internal and external quality controls, along with the integration of LINE-1 hypomethylation with other biomarkers, will be crucial to improving accuracy and realizing its full potential as an important tool in precision oncology.

## Figures and Tables

**Table 1:** LINE-1 cfDNA methylation assessment in various cancers and disease indications.

Cancer	Assay	Clinical Use	Main Finding(s)	Ref.
Breast cancer	MSRE-PCR	Diagnostic	LINE-1 methylation in cfDNA was significantly lower in BC patients (n = 26) than in healthy controls (n = 36) with an AUC of 0.7808 (*p* = 0.01).	[90]
Breast cancer	MS-PCR	Diagnostic	LINE-1 methylation was significantly lower in BC patients (n = 64) compared to healthy individuals (n = 64) with an AUC of 0.89, a sensitivity of 78%, and a specificity of 83% at the cut-off value ≤ 90.	[97]
Lung Cancer	MS-PCR	Diagnostic	LINE-1 methylation was significantly lower in lung cancer patients (n = 64) compared to healthy individuals (n = 64) with an AUC of 0.848, a sensitivity of 75%, and a specificity of 87.50% at a cut-off point of ≤90.	[97]
Lung cancer	Affinity capture coupled with qPCR and targeted sequencing	Diagnostic	LINE-1 promoter methylation index was significantly lower in lung cancer patients (n = 56) than in healthy individuals (n = 44) with an AUC of 0.69 (*p* = 0.0012).	[117]
Lung cancer	MS-PCR	Diagnostic	cfDNA LINE-1 methylation levels in lung cancer patients (n = 16) were significantly lower than in the control group (n = 23), including healthy donors and patients with bronchitis or chronic obstructive pulmonary disease (AUC of 0.83).	[98]
Colorectal cancer	MS-PCR	Diagnostic	Patients with early-stage, as well as advanced disease (n = 114), had significantly higher cfDNA methylation index (e.g., increased LINE-1 hypomethylation) than healthy controls (n = 53), with AUC values of 0.79 and 0.83 in stage I/II and stage III/IV CRC patients, respectively.	[99]
Colorectal cancer	Pyrosequencing	Diagnostic	Decreased LINE-1 methylation in cfDNA was observed in colon adenomas (n = 14) and CRC (n = 13) cases compared to healthy controls (n = 10). At a cut-off of 80.0%, LINE-1 methylation distinguished adenomas from controls with 66.7% sensitivity, 90.0% specificity, and an AUC of 0.8 (*p* < 0.05).	[112]
Prostate cancer	Quantitative pyrosequencing	Diagnostic	LINE-1 methylation was lower in the cfDNA of patients (n = 27) compared to that of healthy individuals (n = 24). Plasma but not tissue LINE-1 methylation was associated with a higher Gleason score (≥7).	[113]
Esophageal adenocarcinoma	MSRE-PCR	Diagnostic	Patients with esophageal adenocarcinoma (n = 19) had hypomethylated LINE-1 in their cfDNA. Longitudinal analysis in two patients with Barrett’s esophagus suggested a correlation between LINE-1 methylation status in cfDNA and progression to esophageal adenocarcinoma.	[91]
Gastric cancer	A variant of MSRE-PCR	Prognostic	Recurrence-free survival and overall survival of gastric patients (total n = 90) with low LINE-1 methylation before surgery were worse than those with high methylation levels (*p* = 0.08 and *p* = 0.11, respectively).	[92]
Hepatocellular carcinoma	Combined bisulfite restriction analysis PCR	Prognostic	LINE-1 hypomethylation in serum was higher in patients (n = 85) than in controls (n = 50) and associated with increased tumor size and advanced tumor stages and proved to be a significant and independent prognostic factor of overall survival in HCC.	[106]
Hepatocellular carcinoma	MS-PCR	Prognostic	LINE-1 hypomethylation in serum was significantly associated with poor survival of HCC patients (n = 50).	[102]
Hepatocellular carcinoma	Pyrosequencing	Prognostic	HCC patients (total n = 172) with plasma LINE-1 methylation levels below 70% had significantly worse overall survival compared to those with levels at or above 70%.	[114]
Hepatocellular carcinoma	MS-PCR	Prognostic	LINE-1 hypomethylation was higher in patients (n = 105) than in controls (n = 50) and associated with surface antigen positivity of the Hepatitis B virus, tumor size, and alpha-fetoprotein levels. LINE-1 hypomethylation and RASSF1A promoter hypermethylation were associated with early recurrence and poor prognosis after curative resection.	[103]
Malignant melanoma	MS-PCR	Prognostic	Patients (total n = 56) who harbored either serum unmethylated LINE-1 or methylated *AIM1* had shorter survival compared with patients with neither.	[105]
Lung cancer	MS-PCR	Evaluation of neoadjuvant chemotherapy and surgery	LINE-1 methylation levels in the cell surface-bound cfDNA fraction increased 2-fold after chemotherapy and 3-fold after surgery, with a stronger effect in patients (total n = 16) with squamous cell lung cancer compared to those with adenocarcinoma.	[104]
Rectal cancer	MS-PCR	Prediction of therapy response	To evaluate methylation markers in cell surface-bound cfDNA during long-term follow-up of rectal cancer patients (n = 25), serial cfDNA analysis showed that patients with relapses had increased SEPTIN9 and IKZF1 methylation and decreased LINE-1 methylation compared to levels 10–15 days post-surgery.	[100]
Colorectal cancer	MS-PCR	Monitoring outcomes of systemic chemotherapy	LINE-1 methylation declined while gene promoter methylation and homocysteine levels increased in the patients ( total n = 55) with progressive disease receiving chemotherapy, with opposite changes in cases with remission.	[101]
Breast and lung cancers	Methylation-related targeted sequencing	Prediction of the effectiveness of ICB therapy	Significant LINE-1 methylation loss in plasma was observed during tumor progression in a breast cancer (n = 91) cohort. A cfDNA-based methylation assay effectively predicted disease progression under ICB therapy. In lung cancer patients (total n = 167), non-responders to ICB showed progressive LINE-1 hypomethylation in cfDNA during early treatment (3 and 6 weeks), while responders exhibited progressive hypermethylation.	[118]

Abbreviations: MS-PCR, methylation-specific PCR; MSRE-PCR, methylation-sensitive restriction enzyme-based PCR.
